# Primary hepatic diffuse large B- cell lymphoma mimicking cholangiocarcinoma

**DOI:** 10.1186/s41824-024-00215-7

**Published:** 2024-08-01

**Authors:** Taruna Yadav, Deepanksha Datta, Poonam Elhence, Vaibhav Varshney, Rajesh Kumar

**Affiliations:** 1 Department of Diagnostic and Interventional Radiology, All India Institute of Medical Sciences, Jodhpur, India; 2 Department of Nuclear Medicine, All India Institute of Medical Sciences, Jodhpur, India; 3 Department of Pathology and Lab Medicine, All India Institute of Medical Sciences, Jodhpur, India; 4 Department of Surgical Gastroenterology, All India Institute of Medical Sciences, Jodhpur, India

**Keywords:** Hepatic lymphoma, Diffuse large B – cell lymphoma, F-18 FDG PET/CT

## Abstract

Primary lymphoma of liver is a rare malignancy with non-specific clinical features and tumor markers. The presentation and imaging features may be indistinguishable from other hepatic malignant lesions. Pathological diagnosis is the gold standard, and early detection is essential to choose the treatment modality. Here, we share an interesting case of Primary Diffuse Large B cell lymphoma of liver and its imaging findings on Computed tomography (CT), Magnetic Resonance Imaging (MRI) and F-18 FDG PET/CT.

## Introduction

Hepatic lymphoma is more commonly secondary in nature due to systemic lymphoproliferative involvement. The Primary hepatic lymphoma is a rare disease that should be considered in the differential diagnosis of other primary hepatic malignancies, particularly cholangiocarcinoma, on cross-sectional imaging (Memeo et al. 1999). With this case, we share the imaging findings of this rare primary liver malignancy and highlight the pivotal role of F-18 FDG (2-fluoro 2-deoxy D glucose) PET/CT (positron emission tomography/ computed tomography) in its diagnosis.

## Case report

**Fig. 1:**
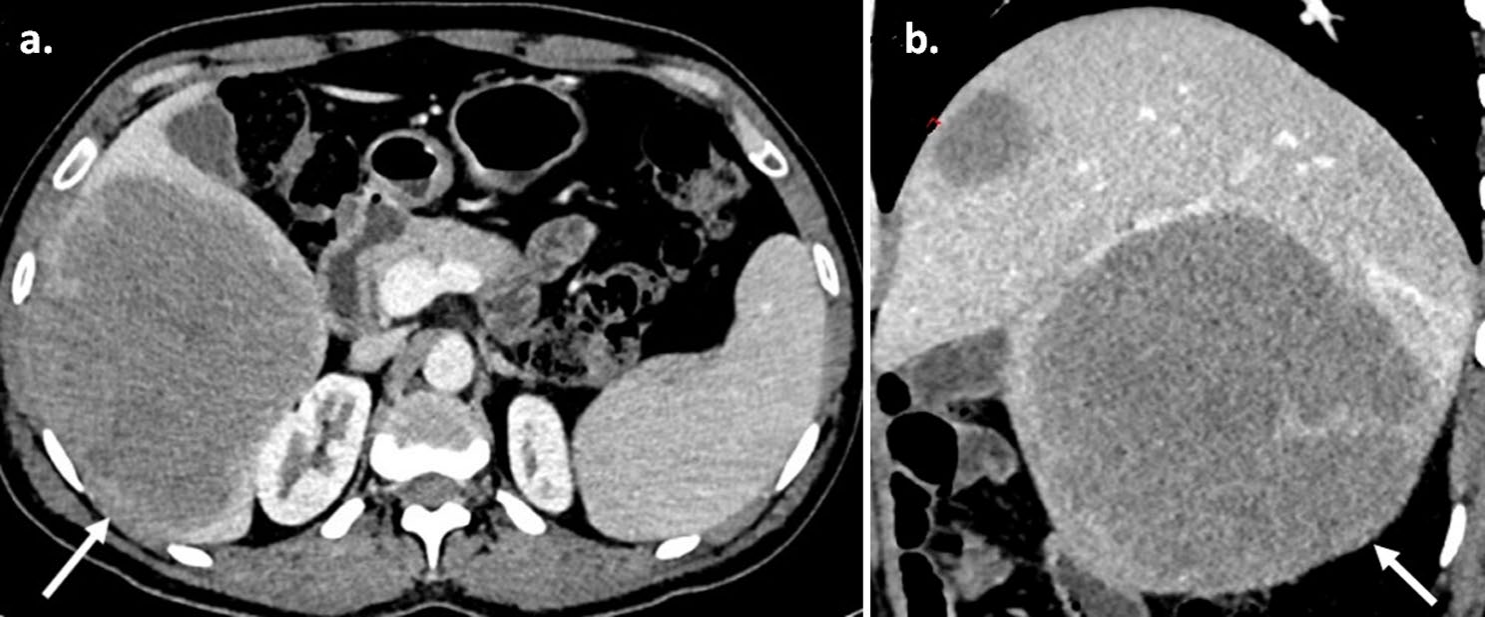
Contrast enhanced CT of liver. (**a**) Axial, (**b**) Sagittal CT images showing two hypoenhancing lesions (white arrows) with absence of perilesional edema

**Fig. 2:**
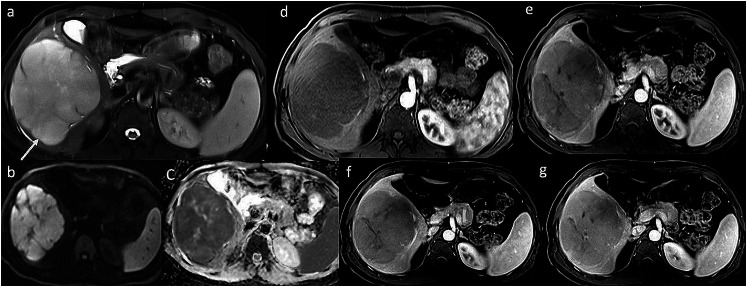
Multiphasic contrast enhanced MRI of liver. (**a**) Axial T2W image shows a hyperintense lesion (arrow) which depicts marked diffusion restriction on diffusion weighted image (**b**) and corresponding apparent diffusion coefficient (ADC) map (**c**) suggesting high cellularity of the lesion. Post-contrast T1W images in (**d**) early arterial, (**e**) late arterial, (f) portal venous, (g) hepatic venous phases show hypoenhancing lesion with mild progressive enhancement of the lesion

**Fig. 3:**
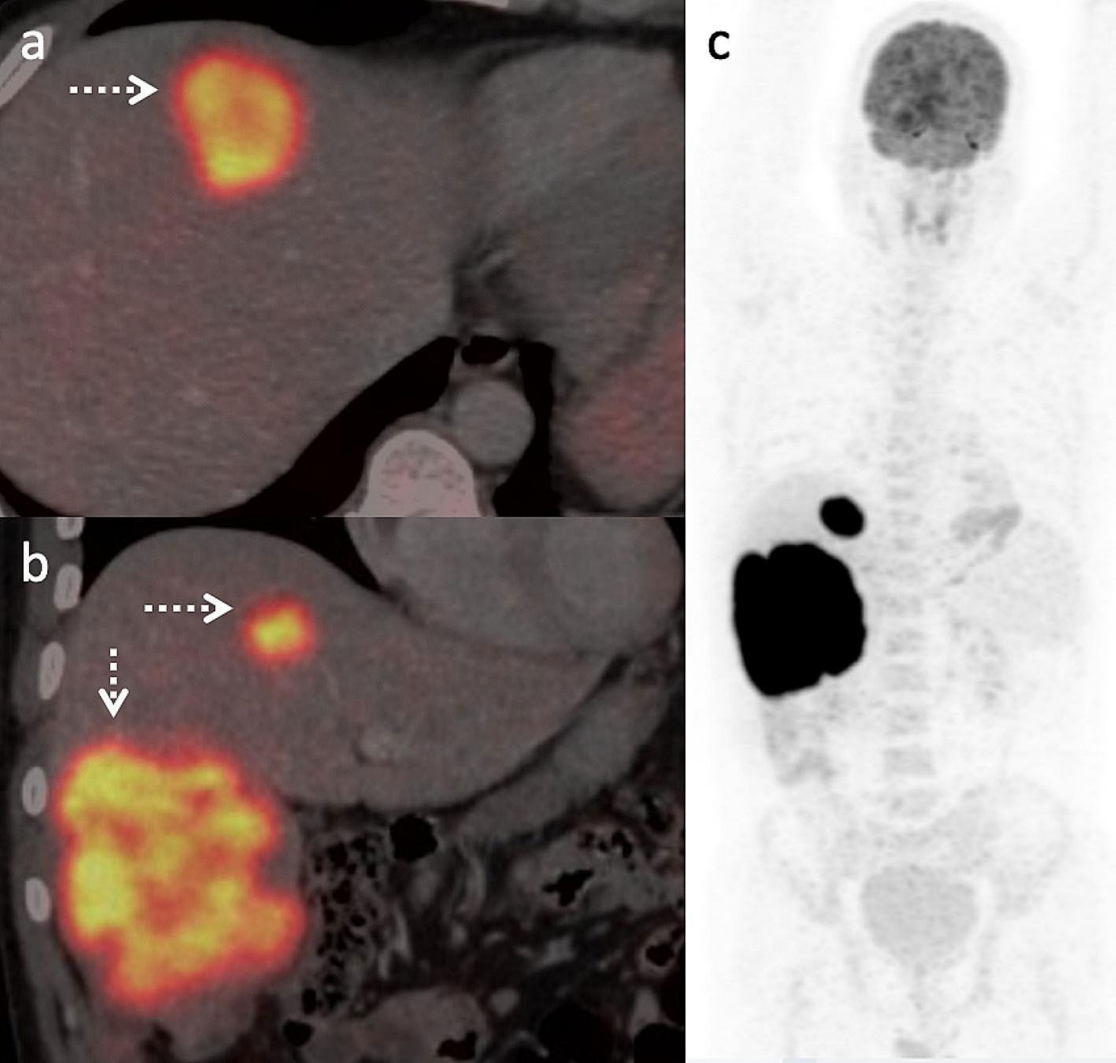
Fused axial (**a**) and coronal (**b**) along with maximum intensity projection (**c**) images of F-18 FDG PET/CT showed intense metabolism in these hepatic lesions (arrows)

**Fig. 4:**
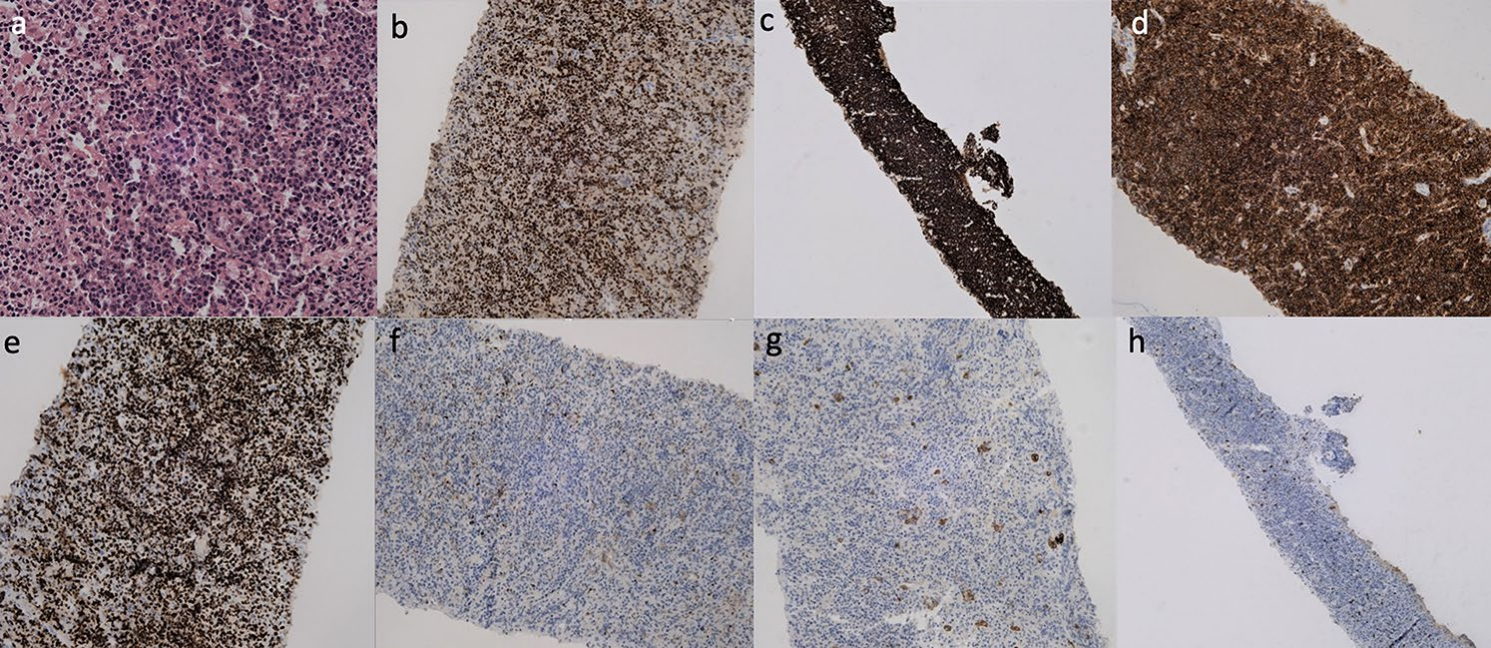
Germinal centre B-cell like Diffuse Large B- cell Lymphoma. **a**) *Hematoxylin and Eosin staining* (H&E), 400x image shows diffuse infiltration of atypical lymphoid cells exhibiting nuclear pleomorphism. Immunohistochemistry revealed membranous expression of Bcl6 (**b**), CD 20 (**c**) and CD 10 (**d**) with high Ki 67 index (> 90%, **e**). It was immunonegative for Bcl2 (**f**) and Pancytokeratin (**g**) with few interspersed T cells on CD3 (**h**)

A 37-year-old male presented with dragging sensation and mild pain in the right hypochondrium associated with weight loss for 1 month. Contrast-enhanced CT axial and sagittal images (Fig. 1; a-b) showed three hypo-enhancing hypodense lesions in the segments IV, V- VI and VII of the liver and absence of perilesional edema and regional lymph nodes with normal liver background. MRI of the liver (Fig. 2; a-g) showed T2 hyperintense lesions with marked restriction on the diffusion-weighted image and the corresponding apparent diffusion coefficient map suggesting high cellularity. Maximum Intensity Projection, fused axial and coronal images of F-18 FDG PET/CT (Fig.3; a-c) showed intense metabolic activity (SUV max- 20) in these liver lesions. There was no metabolically active disease elsewhere in the body. Serum Alpha-fetal protein, Cancer Antigen 19.9 (CA 19.9) and bilirubin were normal. Hepatitis (B and C) viral cytology was negative. Hepatic enzymes were raised with serum aspartate aminotransferase (AST), alanine transaminase (ALT) and alkaline phosphatase (ALP) being 86 U/l, 110 U/l and 152 U/l respectively. Serum lactate dehydrogenase was also elevated (410 U/l). Based on the CT, MRI, PET/CT findings a diagnosis of primary hepatic lymphoma was considered more likely than the intrahepatic cholangiocracinoma. Ultrasound guided biopsy of the liver lesion was performed. The histopathological examination and subsequent immunohistochemistry of the liver lesion (Fig. 4; a-h) confirmed the diagnosis of Germinal centre B-cell like Diffuse Large B- cell Lymphoma.

## Discussion

Primary Non-Hodgkin’s lymphoma of liver is a rare disease (Memeo et al. 1999) with poor prognosis (Freeman et al. 1972). The absence of lymphadenopathy and extra-hepatic lesions differentiate it from the secondary hepatic involvement in lymphoproliferative disorders (Patel et al. 2015).Its risk factors include viral infections like HIV, HBV, HCV and EBV (Santos et al. 2003), immunosuppressive therapy (Rostaing et al. 1995) and cirrhosis (Goldin et al. 1993). Its primary treatment remains chemotherapy (Murthy et al. 2000), however surgery, radiotherapy and combined modalities have been documented (Daniel et al. 1985; Pescovitz et al. 1990; Page et al. 2001). On CT, the differential diagnoses include other primary hepatic tumors (hepatocellular carcinoma, cholangiocarcinoma, focal nodular hyperplasia), hepatic metastases and systemic lymphoma. Though literature is limited, a T2 hyperintense lesion with an intense diffusion restriction on MRI and focal or diffuse FDG uptake on PET/CT with absence of other systemic (extra-hepatic) and nodal involvement is documented in few reports (Basheer et al. 2022; Mahajan et al. 2016; Seshadri et al. 2010; Bohlok et al. 2018).The space-occupying lesions in the liver that show high metabolic activity on F-18 FDG PET/CT include metastases, lymphoma (systemic), cholangiocarcinoma and poorly differentiated hepatocellular carcinoma or neuroendocrine carcinoma (Rachh and Basu 2014). Differential diagnosis in our case was intrahepatic cholangiocarcinoma due to its multi-focal, hypovascular nature in a non-cirrhotic liver (Seo et al. 2017). However, the absence of biliary dilatation, vascular invasion, capsular retraction with presence of marked diffusion restriction , intense metabolic activity in the liver lesions and normal serum CA19.9 were against this diagnosis. In this case, we highlight the imaging of primary hepatic lymphoma in the differential diagnoses of space-occupying lesions of the liver with normal tumor markers and elevated serum lactate dehydrogenase. (see Figs. 3, 4). 

## Conclusion

The hybrid F-18 FDG PET/CT is of significant importance in differentiating primary hepatic lymphoma from other primary malignancies of the liver due to its intense metabolic activity.

## Data Availability

Not applicable.
